# Nonvolatile taste compounds of Shanghai smoked fish: A novel three stages control techniques

**DOI:** 10.1002/fsn3.1960

**Published:** 2020-11-24

**Authors:** Yu Zhou, Shunsheng Chen, Xichang Wang, Hongcai Zhang

**Affiliations:** ^1^ Laboratory of Aquatic Products Quality & Safety Risk Assessment (Shanghai) at China Ministry of Agriculture Shanghai Ocean University Shanghai China; ^2^ College of Food Science and Technology Shanghai Ocean University Shanghai China; ^3^ National R&D Branch Center for Freshwater Aquatic Products Processing Technology (Shanghai) Shanghai Ocean University Shanghai China; ^4^ School of Agriculture and Biology Shanghai Jiao Tong University Shanghai China

**Keywords:** ATP‐related compounds, free amino acids, grass carp, nonvolatile flavor

## Abstract

In this work, the effect of processing stages including first soaking (FS), frying after first soaking (FFS), and second soaking (SS) on nonvolatile taste compounds of Shanghai smoked fish was investigated using high‐performance liquid chromatography (HPLC) and automatic amino acid analyzer. Results showed that the contents of free amino acids (FAAs) ranged from 396.94 to 585.79 mg/100 g and 5′‐inosine monophosphate (IMP, as main umami nucleotide) from 215.91 to 284.56 mg/100 g in Shanghai smoked fish, respectively. Moreover, the contents of Glu and Gly as main umami amino acids ranged from 1.64 to 107.32 mg/100 g and 61.61 to 108.88 mg/100 g, respectively. TAV values of IMP, Asp, and Glu in Shanghai smoked fish reached 11.38, 2.73, and 21.46, respectively. The obvious difference could be observed using principal component analysis (PCA) in three processing stages of Shanghai smoked fish. Therefore, probing into the nonvolatile flavor of Shanghai smoked fish could not only enrich the theoretical basis of flavor chemistry in freshwater fish fields, but probe into the formation mechanisms of taste compounds in further study.

## Graphical abstract

Removing the native unpalatable taste of grass carp is key for increasing its umami flavor and economic value. Therefore, the effect of processing stages including first soaking (FS), frying after first soaking (FFS), and second soaking (SS) on nonvolatile taste compounds of Shanghai smoked fish was investigated using high‐performance liquid chromatography (HPLC) and automatic amino acid analyzer.

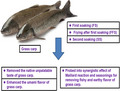

**Highlights**

- First report about the nonvolatile taste compounds of Shanghai smoked fish.
- Three processing stages including FS, FFS, and SS were used for preparing the Shanghai smoked fish.
- Synergetic effect of Maillard reaction and seasonings on tastes of Shanghai smoked fish was investigated.
- Earthy‐musty tastes of grass carp were covered up as well as umami flavor was increased.
- Electronic tongue could effectively distinguish the taste profiles of Shanghai smoked fish.

## 1 INTRODUCTION

Grass carp (*Ctenopharyngodon idellus*), together with black carp, silver carp, and bighead carp, are known as "the four major Chinese carps." Grass carp with abundantly nutritional values is one of the largest freshwater fish in China, accounting for approximately 71% of all freshwater aquaculture production (Xie et al., 2018). In 2018, the output of grass carp reached 55,043 million tons accounting for about 21.63% and ranking the first in freshwater fish (Xu, Wu, Guo, 2019). Grass carp usually grows in freshwater such as pond or lake, but bacteria with earthy smell are easily attached to plankton such as diatom and cyanobacteria, leading to accumulation of bad odor substances through food chain in grass carp (Zhong et al., 2011; Zhou & Wang, 2006). The earthy‐musty compounds mainly include 2‐methylisoborneol (2‐MIB), geosmin, trimethylamine, and hexanal (Robertson et al., 2005; Yang, Hu, et al., 2019; Yang, Shi, et al., 2019; Zhao, Wu, et al., 2015; Zhao, Shen, et al., 2015; Zhao, Zou, et al., 2015). Thus, removing the native unpalatable taste of grass carp is key for increasing its umami flavor and economic value.

In recently years, the deodorization methods of aquatic products consist of three categories: physical deodorization methods (embedding, adsorption, salt dissolution, masking, organic solvent extraction, irradiation, etc), chemical deodorization (acid‐base and antioxidant method), and biological deodorization (Hong et al., 2013; Yarnpakdee, et al., 2012; Fukami, 2006.; Hu & Pan, 2000; Kuley et al., 2012; Tomac et al., 2015; Giorgio et al., 2019). Physical deodorization methods include low‐cost of natural zeolite and active carbon adsorption, but time‐consuming and low efficiency limited their wide application (Kuley et al., 2012). Irradiation has not been widely used in aquaculture based on the consideration of food safety (Tomac et al., 2015). Although liquid‐liquid extraction was effective in fish oil deodorization at high‐temperature condition (70°C) (Song, Zhang, et al., 2018; Song, Wang, et al., 2018), the use of organic solvents resulted in damage of fish compositions and food safety problems was also the pitfall of this method.

Maillard reaction (MR) refers to the process that carbonyl compounds (reducing sugars) and amino compounds (amino acids and proteins) are polymerized and condensed into melanoids at certain conditions. It is particularly important for the formation of meat flavors (Dong et al., 2019; Thomas & Josephine, 1959; van den Ouweland, Demole & Enggist, 1989). Priazines, alcohols, and aldehydes, as the major flavor contributors, accounted for 38.86, 9.21, and 8.23% aroma of Maillard reaction products (MRPs) from *Collichthys niveatus* (*C. niveatus*) protein hydrolysates (Zhao, Wu, et al., 2015; Zhao, Shen, et al., 2015; Zhao, Zou, et al., 2015). The contents of total free amino acids (FAAs) increased from 53.36 nmol/kg in fresh grass carp to 65.03 and 72.18 nmol/kg in fried samples for 2 and 4 min, respectively, indicating frying significantly contributed to improving the taste and flavor compounds of grass carp (Li, Tu, Sha, et al., 2016; Li, Tu, Zhang, et al., 2016). Previous report also indicated that fishy smell in grass carp soup could be improved by masking function of garlic and ginger, known as "sensational deodorizing" (Li, Tu, Sha, et al., 2016; Li, Tu, Zhang, et al., 2016). Therefore, it was hypothesized that the synergistic effect of MR and seasonings could effectively remove fishy and earthy flavor of grass carp.

Shanghai smoked fish deeply loved by public is a traditional special dish with crispy crust and delicious taste. The attractive flavor of grass carp could be increased with the help of MR and seasonings. Wang et al., previously investigated the contents of free amino acids (FAAs), ATP‐related compounds (ATPs) of dead grass carp stored at 4°C for 12 hr (Wang et al., 2018). Few study focused on the nonvolatile flavor compounds of Shanghai smoked fish by adjusting the conditions of MR and the addition ratios of seasonings.

Therefore, the present study was designed to study on the effects of first soaking (FS), frying after first soaking (FFS) and second soaking (SS) on the taste compounds of Shanghai smoked fish. A detailed study on the water‐soluble compounds of Shanghai smoked fish could not only enrich the theoretical knowledge of flavor chemistry of freshwater fish, but have a profound contribution to the development of freshwater fish processing techniques.

## 2 MATERIALS AND METHODS

### 2.1 Materials

Standards of adenosine triphosphate (ATP), adenosine diphosphate (ADP), 5′‐inosine monophosphate (IMP), and hypoxanthine (Hx) were purchased from Sigma. Standards of 5′‐adenosine monophosphate (AMP) and hypoxanthine riboside (HxR) were purchased from Tokyo Chemical Industry (TCI) Co., Ltd., Japan. KH_2_PO_4_, K_2_HPO_4_ and methanol (chromatographic grade), perchloric acid, KOH, trichloroacetic acid (TCA), and NaOH were from Sinopharm Chemical Reagent Co., Ltd, Shanghai, China. The 17 amino acids mixtures (chromatographic grade) were bought from National Institute of Metrology, China‐Institute of Stoichiometry and Analytical Sciences, Beijing, China.

The live grass carps (about 2.5 kg/tail), chive, ginger, cooking wine (Wangzhihe Food Co., Ltd), salt (China Salt Co., Ltd), pepper (Haoshihui condiment Co., Ltd), soy sauce (Haitian seasoning Food Co., Ltd), sunflower seed oil (Jinlongyu Food Co., Ltd), five‐spice powder (Weihaomei Food Co., Ltd), and sugar (Fengyiyuan Food Co., Ltd) were purchased from NGS Supermarket (No.570, Guzong Road, Pudong New Area).

### 2.2 Preparation of Shanghai smoked fish

Grass carps alive were timely stunned, killed, scaled, gutted, rinsed, and filleted (length × width × height, 9 × 1.5 × 1.5 cm) after transporting to the laboratory. Seasonings formula of preparing Shanghai smoked fish is shown in Table 1. Processing stages of Shanghai smoked fish could be divided into three stages including FS, FFS, and SS. The detailed treatment process was shown below. (a) grass carp was soaked in the seasoning mixtures including cooking wine (20%), salt (1.5%), chive (2%), ginger (1%), garlic (1%), pepper (1%), and soy sauce (5%) for 30 min. (b) grass carp after FS treatment was fried on the electric frying pan (HY‐81, Nanhai Poffey Electrical and Mechanical Equipment Co., Ltd.) at 180°C for 5 min. (3) Grass carp after FFS treatment was soaked for 10 min in boiling water (60%) containing sunflower seed oil (5%), five‐spice powder (0.1%), sugar (3%), and soy sauce (10%) in nonstick pot (Hertz Electric Appliances Co., Ltd.). Both liquid and oil of soaking and frying fish fillets were drained by kitchen paper.

**Table 1:** Seasonings formula of preparing Shanghai smoked fish

Treatment process	Formula								
FS	A_0_	A_1_	A_2_	A_3_	A_4_	A_5_	A_6_	A_7_	A_8_
FFS	B_0_	B_1_	B_2_	B_3_	B_4_	B_5_	B_6_	B_7_	B_8_
SS	C_0_	C_1_	C_2_	C_3_	–	–	–	–	–

Note

A_0_–A_8_: no adding; cooking wine; cooking wine + salt; cooking wine + chive; cooking wine + ginger; cooking wine + garlic; cooking wine + pepper; cooking wine + soy sauce; all seasonings.

B_0_–B_8_: no adding; cooking wine; cooking wine + salt; cooking wine + chive; cooking wine + ginger; cooking wine + garlic; cooking wine + pepper; cooking wine + soy sauce; all seasonings.

C_0_–C_3_: all seasonings + plant oil; all seasonings + plant oil + sugar; all seasonings + plant oil + sugar +soy sauce; all seasonings + plant oil + sugar +soy sauce + five‐spice powder.

All seasonings represented cooking wine + salt +chive + ginger +garlic + pepper +soy sauce.

Abbreviations: FFS, frying after first soaking; FS, first soaking; SS, second soaking.

### 2.3 ATP‐related compounds (ATPs) analysis of Shanghai smoked fish

ATP‐related compounds were determined based on previous procedures described by Yokoyama et al. (Yokoyama et al., 1992). The 5 g of fish fillets was accurately weighed before adding 10 ml of cold perchloric acid (10%, v/v), and then centrifuged (H2050R, Xiangyi Co., Ltd.) at 10,000 rpm at 4°C for 15 min after homogenization for 2 min. The same treatment process was repeated twice except for adding 5 ml of cold perchloric acid (5%, v/v).

The collected supernatants were combined and adjusted to pH 6.50 with 1 mol/L KOH, and then stood for 30 min. The supernatants were diluted to 50 ml, shaken and filtered through a 0.22 μm membrane. All operating conditions were conducted at 4°C.

Amounts of ATPs were determined by high‐performance liquid chromatography (HPLC) (W2690/5, Waters Co., Ltd) according to previous report (Qiu et al., 2015). ODS‐SPC^18^ column (4.6 mm × 250 mm, 5 μm) was employed for the separation of ATPs at 28°C. The mobile phase A and B were 0.05 mol/L KH_2_PO_4_ and K_2_HPO_4_ (1:1, v:v), and methanol, respectively. To improve the separation efficiency of ATPs, the flow rate and detection wavelength was set as 1.0 ml/min and 254 nm, respectively.

### 2.4 Free amino acids (FAAs) analysis of Shanghai smoked fish

The concentration of FAAs was analyzed by an automatic amino acid analyzer (L‐8800, Hitachi) based on previously reported method with slight modifications (Jia et al., 2017). Samples (2 g) were homogenized with 15 ml of 15% TCA and stood for 2 hr, then centrifuged at 10,000 rpm at 4°C for 15 min. The obtained sediment (5 ml) was adjusted to pH 2.0 with 3 mol/L NaOH and diluted to 10 ml before filtration through a 0.22 μm filter membrane.

### 2.5 Electronic tongue analysis of Shanghai smoked fish

Samples (2 g) were homogenized with 25 ml ultrapure water, and stood for 15 min, followed by centrifugation at 10,000 rpm for 10 min at 4°C. The sediment was filtered through a 0.22 μm membrane for further analysis (Yang, Hu, et al., 2019; Yang, Shi, et al., 2019). Samples were diluted 80 times before Electronic tongue (Alpha M.O.S., Astree, France) analysis based on previously reported conditions (Yang, Hu, et al., 2019; Yang, Shi, et al., 2019). The 120 s response value on each sensor was selected as the original data of electronic tongue.

To prevent the carryover effects, ultrapure water was used for cleaning after test and cleaning period was 10 s. Each sample was determined 8 times and the last three data were analyzed by principal component analysis (PCA).

### 2.6 Calculation of taste activity value (TAV)

TAV represented the ratio of the content of flavor substances in samples to their corresponding taste threshold.

(1) $$\text{TAV}=\frac{\text{The ratio of the content of flavor substances}}{\text{Corresponding taste threshold}}$$

where TAV > 1 means this substances contributed significantly to flavor and TAV < 1 means not significant (Gunlu & Gunlu, 2014).

### 2.7 Statistical analysis

All data were represented by means ± standard deviations (*SD*) of three independent replicates. The data analysis was performed using Microsoft Excel 2010 and SPSS Statistics 17.0. The Duncan's test of one way ANOVA was used for significant differences analysis. Original data were collected and analyzed by PCA after electronic tongue analysis.

## 3 RESULTS AND DISCUSSION

### 3.1 Effects of FS on ATPs contents

Inosine monophosphate, GMP, and AMP are key components of strong umami taste (Chen & Zhang, 2007; Johnson et al., 2013). As shown in Table 2, results showed that IMP contents of A_0_–A_8_ were 215.91, 156.59, 180.05, 164.91, 175.84, 200.32, 199.48, 275.57, and 270.20 mg/100 g, respectively. Among them, IMP contents of cooking wine and soy sauce soaking sample were the lowest and highest, respectively. Although the contents of AMP in each sample were low, AMP has synergistic effects with IMP, Glu, and Asp on increasing umami of Shanghai smoked fish (Zhang et al., 2019). IMP contents of samples decreased significantly after soaking in cooking wine, but increased significantly after soaking in soy sauce. TAV of IMP in all samples after FS treatment were greater than 1, among them, soy sauce soaking sample was 27.55%, higher than that of fresh fish, indicating soy sauce and other seasonings contributed to the tastes of Shanghai smoked fish (Lioe et al., 2010). The main reason was that cooking wine with organic acids decreased the pH of fish (Lin et al., 2012; Zhao, Wu, et al., 2015; Zhao, Shen, et al., 2015; Zhao, Zou, et al., 2015), which promoted the degradation of IMP (Thanh & Peter, 1985). In addition, Asp and Glu were recognized as main synthesis substances of ATPs in soy sauce, that is, IMP was synthesized at first, and then converted into AMP and GMP (Lin et al., 2015; Moffatt & Ashihara, 2002). Studies have shown that IMP with strong umami could not only increase sweetness and meat flavor, but inhibit salty, bitter, and burnt flavor of fish. Therefore, IMP was the main umami nucleotide in FS process (Huang et al., 2019).

On the contrary, Hx could interact with certain amino acids and peptides, resulting in little bit of bitterness in Shanghai smoked fish and thus causing undesirable smells (Qiu et al., 2015). The Hx contents in fresh grass carp (A_0_: 15.13 mg/100 g) were higher than other FS samples (A_1_–A_8_: 9.20, 3.41, 3.20, 3.41, 3.31, 3.88, 3.91, and 4.31 mg/100 g, respectively), indicating masking function of seasonings on unfavorable taste compounds of grass carp.

**Table 2:** Contents of ATP‐related compounds in first soaking and frying for preparing Shanghai smoked fish (mg/100 g)

Samples	ATP	ADP	AMP	TAV	IMP	TAV	Hx	HxR
A_0_	15.38 ± 2.94^c^	14.32 ± 2.06^c^	13.54 ± 0.38^e^	0.27	215.91 ± 4.53^c^	8.64	15.13 ± 1.16^d^	20.97 ± 0.21^e^
A_1_	4.73 ± 1.58^ab^	6.76 ± 0.97^b^	7.29 ± 0.81^d^	0.15	156.59 ± 1.68^a^	6.26	9.20 ± 0.23^c^	11.68 ± 0.70^b^
A_2_	2.88 ± 0.92^a^	3.10 ± 0.31^a^	3.48 ± 0.38^a^	0.07	180.05 ± 3.66^b^	7.20	3.41 ± 0.11^ab^	14.28 ± 0.79^c^
A_3_	2.71 ± 0.08^a^	2.73 ± 0.50^a^	2.99 ± 0.03^a^	0.06	164.91 ± 3.28^ab^	6.60	3.20 ± 0.05^a^	8.92 ± 0.38^a^
A_4_	3.20 ± 0.22^ab^	3.14 ± 0.02^a^	3.35 ± 0.02^a^	0.07	175.84 ± 3.34^b^	7.03	3.41 ± 0.07^ab^	12.88 ± 0.22^bc^
A_5_	3.78 ± 0.62^ab^	3.54 ± 0.61^a^	4.54 ± 0.51^b^	0.09	200.32 ± 16.59^c^	8.01	3.31 ± 0.11^a^	14.19 ± 0.73^c^
A_6_	6.02 ± 0.70^b^	6.47 ± 0.21^b^	7.87 ± 0.39^d^	0.16	199.48 ± 4.05^c^	7.98	3.88 ± 0.29^ab^	25.18 ± 1.31^f^
A_7_	5.17 ± 0.20^ab^	5.73 ± 0.58^b^	6.09 ± 0.09^c^	0.12	275.57 ± 11.52^d^	11.02	3.91 ± 0.00^ab^	19.38 ± 0.28^d^
A_8_	5.98 ± 0.74^b^	6.06 ± 0.60^b^	5.96 ± 0.25^c^	0.12	270.20 ± 4.99^d^	10.81	4.34 ± 0.14^b^	24.88 ± 0.78^f^
B_0_	12.31 ± 1.40^jk^	9.54 ± 0.30^ij^	14.91 ± 0.98^i^	0.30	384.93 ± 11.33^k^	15.40	3.89 ± 0.10^i^	28.48 ± 0.46^i^
B_1_	10.46 ± 1.00^i^	11.29 ± 0.63^jk^	15.64 ± 0.24^ij^	0.31	359.19 ± 6.69^jk^	14.37	4.12 ± 0.11^i^	30.45 ± 0.27^j^
B_2_	14.25 ± 0.59^k^	10.97 ± 1.08^ijk^	14.79 ± 0.93^i^	0.30	376.49 ± 14.38^k^	15.06	4.47 ± 0.11^j^	39.09 ± 0.69^m^
B_3_	12.97 ± 0.06^jk^	10.62 ± 0.83^ijk^	17.05 ± 1.16^jk^	0.34	379.17 ± 11.11^k^	15.17	4.55 ± 0.20^jk^	35.37 ± 0.09^kl^
B_4_	11.81 ± 0.72^ij^	10.28 ± 0.06^ijk^	14.73 ± 0.72^i^	0.29	360.29 ± 5.30^jk^	14.41	4.69 ± 0.06^jkl^	34.64 ± 0.86^k^
B_5_	16.75 ± 1.38^l^	11.94 ± 1.37^k^	18.76 ± 0.87^k^	0.38	375.53 ± 7.56^k^	15.02	4.83 ± 0.02^klm^	40.19 ± 1.20^m^
B_6_	12.84 ± 0.59^k^	9.33 ± 0.15^i^	14.42 ± 0.30^i^	0.29	322.25 ± 10.26^i^	12.89	4.90 ± 0.00^lm^	37.17 ± 0.60^l^
B_7_	10.09 ± 1.11^i^	10.72 ± 0.85^ijk^	16.00 ± 1.03^ij^	0.32	337.89 ± 15.77^ij^	13.52	5.04 ± 0.29^m^	34.29 ± 1.09^k^
B_8_	9.14 ± 1.09^j^	11.53 ± 0.03^k^	15.69 ± 0.94^ij^	0.31	370.11 ± 19.58^k^	14.80	4.85 ± 0.10^klm^	35.84 ± 1.23^kl^

Note

A_0_–A_8_: no adding; cooking wine; cooking wine + salt; cooking wine + chive; cooking wine + ginger; cooking wine + garlic; cooking wine + pepper; cooking wine + soy sauce; all seasonings.

B_0_–B_8_: no adding; cooking wine; cooking wine + salt; cooking wine + chive; cooking wine + ginger; cooking wine + garlic; cooking wine + pepper; cooking wine + soy sauce; all seasonings.

All seasonings represented cooking wine + salt +chive + ginger +garlic + pepper + soy sauce.

Different superscript letters (a‐m) in the same column indicate a significant difference (*p* < .05).

Abbreviations: ADP, adenosine diphosphate; AMP, 5′‐adenosine monophosphate; ATP, adenosine triphosphate; Hx, hypoxanthine; HxR, hypoxanthine riboside; IMP, 5′‐inosine monophosphate; TAV, taste activity value.

### 3.2 Effects of FFS on ATPs contents

Table 2 shows the contents of ATPs in FFS of grass carp. Although the contents of AMP in FFS were low, significantly higher than that of FS samples. AMP acting as a good flavor enhancer in aquatic foods could suppress bitterness and produce pleasant sweetness and saltiness (Qiu et al., 2015). IMP contents of B_0_–B_8_ reached 384.93, 359.19, 376.49, 379.17, 360.29, 375.53, 322.25, 337.89, and 370.11 mg/100 g, respectively. IMP contents of FFS with cooking wine and pepper were the lowest, but fried grass carp were the highest (Table 2). Compared with FS samples, the contents of IMP in FFS were increased by 78.28, 129.38, 109.10, 129.93, 104.90, 87.47, 61.55, 22.61, and 36.98% respectively. TAV of IMP of each sample was greater than 12, higher than that of the FS samples (Table 2), showing high‐temperature frying mainly contributed to the tastes of grass carp. The main reason was the accumulation of IMP degraded from ATP by high‐temperature frying (Cui, 2001).

### 3.3 Effects of SS process on the content of ATPs

**FIGURE 1:**
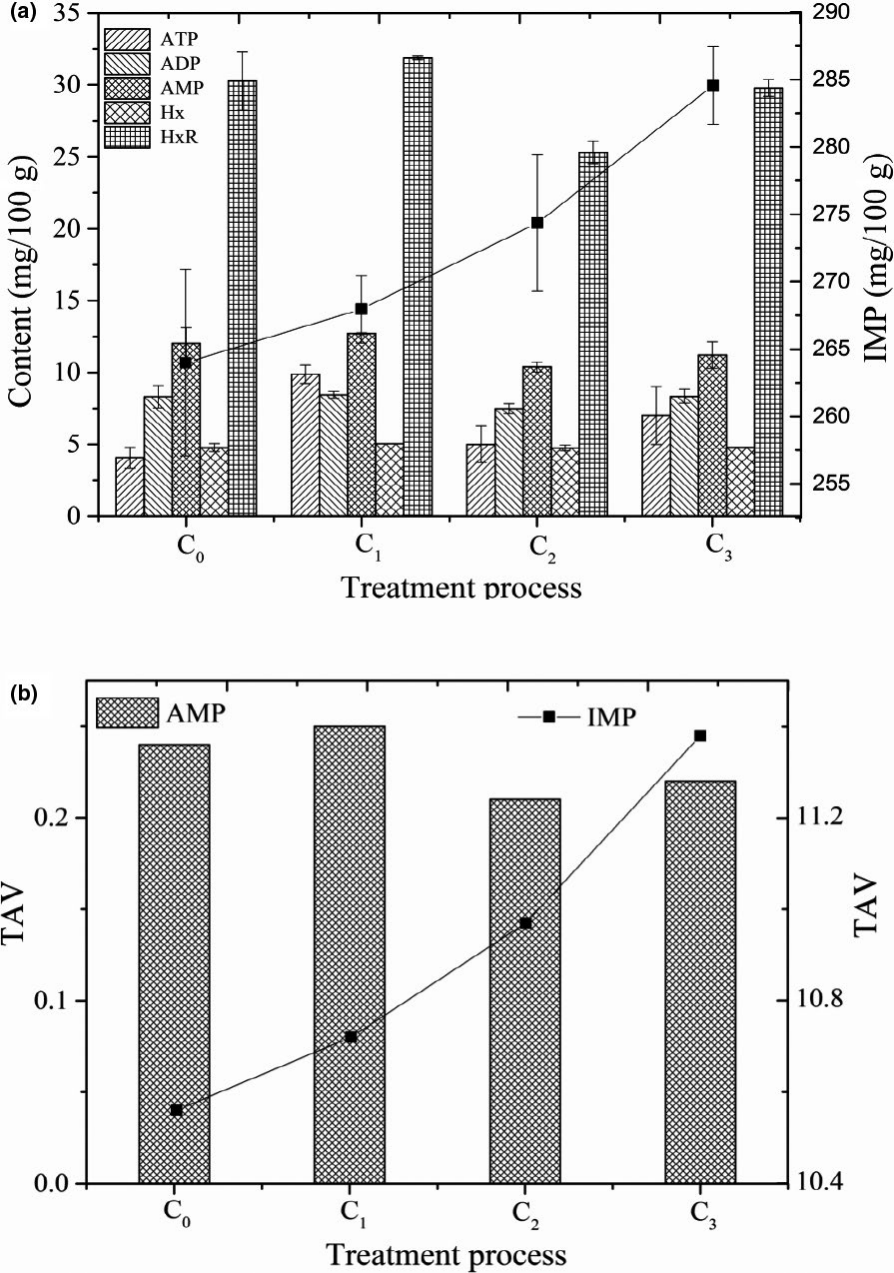
(a) Contents of ATP‐related compounds (mg/100g), and taste activity value (TAV) of AMP and IMP (b) in second soaking for preparing Shanghai smoked fish. C_0_, C_1_, C_2,_ and C_3_ represented all seasonings + plant oil, all seasonings + plant oil + sugar, all seasonings + plant oil + sugar + soy sauce and all seasonings + plant oil + sugar +soy sauce + five‐spice powder, respectively. All seasonings represented cooking wine + salt + chive + ginger + garlic + pepper + soy sauce. ATP: adenosine triphosphate; ADP: adenosine diphosphate; AMP: 5′‐adenosine monophosphate; IMP: 5′‐inosine monophosphate; Hx: hypoxanthine; HxR: hypoxanthine riboside

Second soaking was applied to enhance the tastes of grass carp after FS and FFS treatment. Figure 1a shows the ATPs contents of grass carp were affected by SS process. IMP contents of C_0_–C_3_ were 263.99, 267.96, 274.35, and 284.56 mg/100 g, respectively. IMP contents of SS samples soaked with plant oil and water were the lowest, and that of Shanghai smoked fish final products were the highest (Figure 1a). Plant oil, sugar, and water had no significant effect on IMP content, except for soy sauce. The main reasons were that soy sauce contained the tasting ATPs (Lioe et al., 2010), and Asp and Glu contributed to the synthesis of new IMP, which was consistent with the results in FS‐ and FFS‐treated samples. Moreover, TAV of IMP of each treated sample was more than 10 (Figure 1b), which further indicated that soy sauce was helpful for increasing umami taste of grass carp.

The degradation order of ATP was successively ATP > ADP > AMP > IMP > HxR > Hx (Wang et al., 2018). The certain umami ATPs are also produced with the degradation of ATP. TAV of IMP in A_0_, A_8_, B_8_, and C_3_ was 8.64, 10.81, 14.80, and 11.38, respectively (Table 2 and Figure 1b). Previous studies have shown that IMP had strong umami (Johnson et al., 2013), so it was the main umami ATPs in Shanghai smoked fish, contributing significantly to its tastes.

Contents of AMP were significantly lower than that of IMP. AMP contents of FS samples were the lowest with no significant difference (*p* > .05) compared with other samples (Table 2, Figure 1b). Although the content of AMP in each sample was low, AMP had synergistic effects with IMP, Glu, and Asp (Zhang et al., 2019), leading to savory taste of fish. Bitter Hx has adverse effect on tastes of grass carp, Hx content in Shanghai smoked fish reduced to one third of that in fresh grass carp, indicating that SS treatment could greatly improve the bitterness of grass carp. Three reasons might be accounted for the improvement of savory and palatability taste. (a) ATPs exhibited better stability under neutral conditions than acidic or alkaline conditions (Cui, 2001), meanwhile cooking wine promoted ATP degradation by reducing the pH of matrix. (b) high‐temperature frying accelerated ATPs decomposement with a rapid IMP accumulation (Qiu & Yue, 2016), and (c) taste ATPs was used as umami reinforcing agent after adding soy sauce (Lioe et al., 2010).

**FIGURE 2:**
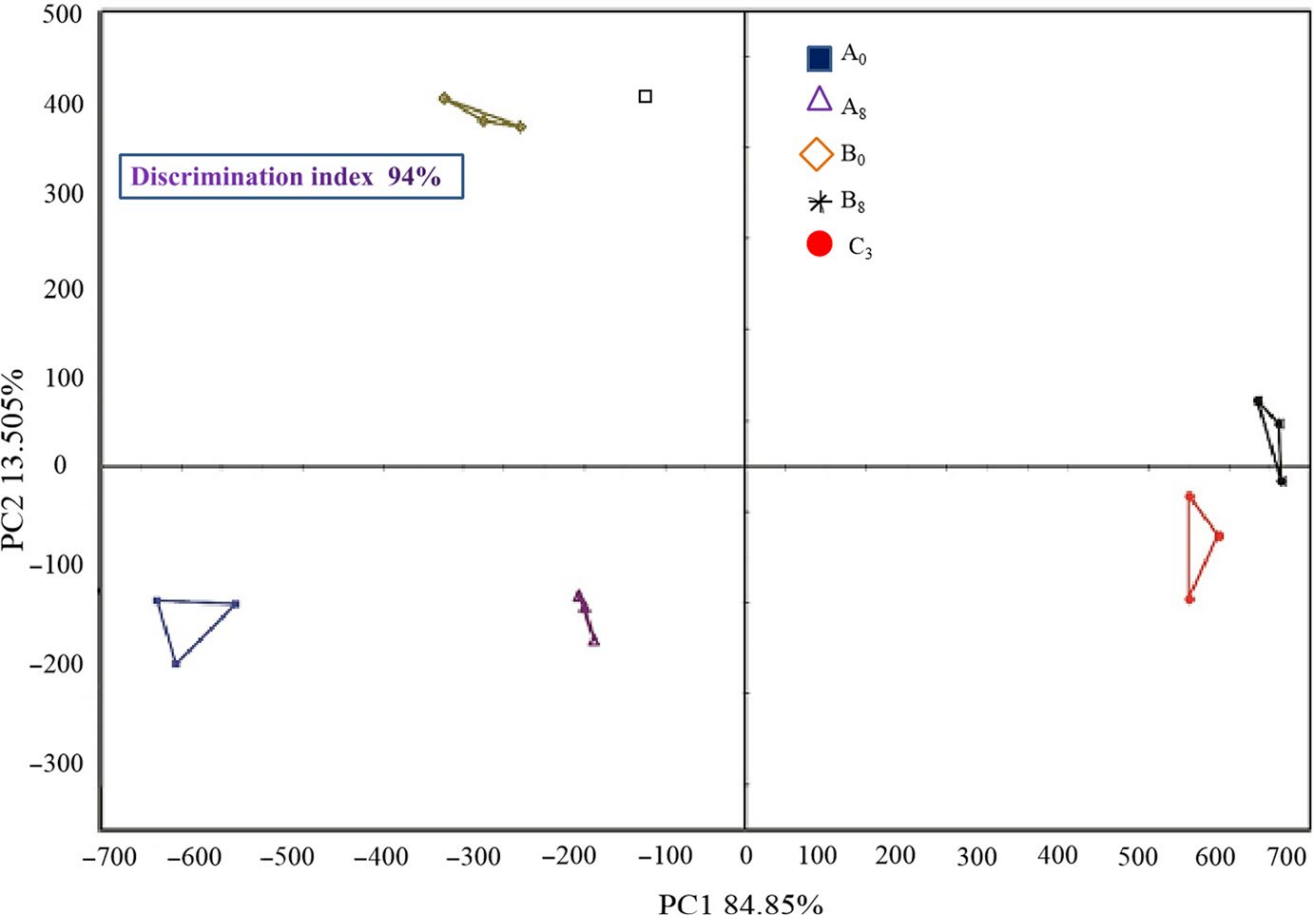
PCA chart of tasting Shanghai smoked fish. A_0_ and B_0_: No adding; A_8_ and B_8_: all seasonings; C_3_: All seasonings + plant oil + sugar + soy sauce + five‐spice powder. All seasonings represented cooking wine + salt + chive + ginger + garlic + pepper + soy sauce

### 3.4 Effects of FS process on the content of FAAs

It is well‐known that FAAs are important water‐soluble flavor components in foods (Chen & Zhang, 2007). They not only had own flavor characteristics, but synergized with other FAAs to produce different taste sensations (Weng, 2011). As shown in Table 3, FAAs contents of A_0_‐A_8_ reached 396.94, 419.54, 427.46, 439.88, 412.05, 418.48, 408.03, 464.44, and 421.02 mg/100 g, respectively, among them, the contents of flavor amino acids accounted for 28.33, 30.64, 33.76, 32.48, 33.08, 29.80, 37.01, 39.98, and 38.66%, respectively. The contents of Phe and Tyr in the two soy sauce soaked samples were higher than that without soy sauce soaking. Phe and Tyr, as important flavor components, were bitter aromatic amino acids, which have been found to be important flavor components in soy sauce in recent years (Chen & Zhang, 2007; Lioe et al., 2010). Contents of flavor amino acid, except for Pro, were significantly higher than that of fresh grass carp, among them, the contents of Asp and Glu were increased after soy sauce soaking (Lioe et al., 2010). Chen & Zhang also showed that Gly, Glu, and Ala contributed to the major sweetness of fish (Chen & Zhang, 2007). The TAV of Glu in fresh grass carp was less than 1, but FS samples were greater than 4 (Table 5). Although the TAV of Asp, Ser, Gly, and Ala were lower than 1 before and after FS, they all increased and had synergistic effect on the flavor of grass carp. The main reasons why FS process could improve the contents of umami were that (a) ethanol in cooking wine (Wang, 2005) and seasonings (salt, chive, ginger, garlic, etc) entered into fish cells through capillaries and intercellular spaces because protein structure was destroyed and promoted the dissolution of amino acids (Pei et al., 2014). (b) Asp and Glu as main FAAs in soy sauce were permeated into fish meat, as well as the decomposition of histones in grass carp.

**Table 3:** Contents of free amino acids in first soaking and frying for preparing Shanghai smoked fish (mg/100 g)

Amino acid types	Taste	Treatment process									Threshold (mg/100 g)
		A_0_	A_1_	A_2_	A_3_	A_4_	A_5_	A_6_	A_7_	A_8_	
Asp*	Umami/sour (+)	0.58 ± 0.02^a^	0.64 ± 0.01^bc^	0.64 ± 0.02^b^	0.64 ± 0.02^b^	0.66 ± 0.02^bc^	0.57 ± 0.03^a^	0.70 ± 0.02^c^	1.45 ± 0.03^d^	1.63 ± 0.04^e^	3
Thr^#^	Sweet (+)	10.07 ± 0.06^bc^	9.91 ± 0.16^ab^	10.85 ± 0.12^d^	10.87 ± 0.17^d^	10.33 ± 0.22^c^	9.54 ± 0.22^a^	10.81 ± 0.10^d^	12.83 ± 0.11^f^	11.54 ± 0.28^e^	260
Ser*	Sweet (+)	4.99 ± 0.18^a^	4.92 ± 0.19^a^	5.77 ± 0.12^b^	5.78 ± 0.19^b^	5.44 ± 0.19^b^	4.73 ± 0.16^a^	6.22 ± 0.13^c^	8.26 ± 0.12^e^	7.66 ± 0.09^d^	150
Glu*	Umami/sour (+)	1.64 ± 0.11^a^	21.10 ± 0.05^b^	23.42 ± 0.15^c^	23.64 ± 0.21^c^	20.98 ± 0.33^b^	23.30 ± 0.23^c^	30.42 ± 0.30^d^	53.79 ± 0.23^f^	41.45 ± 0.71^e^	5
Gly*	Sweet (+)	61.61 ± 0.04^c^	60.30 ± 0.26^b^	69.03 ± 0.16^g^	66.65 ± 0.37^f^	65.26 ± 0.10^e^	54.34 ± 0.27^a^	67.04 ± 0.43^f^	69.15 ± 0.22^g^	63.72 ± 0.17^d^	130
Ala*	Sweet (+)	8.20 ± 0.36^a^	7.83 ± 0.16^a^	10.92 ± 0.10^d^	10.21 ± 0.35^c^	10.00 ± 0.22^c^	9.21 ± 0.25^b^	14.46 ± 0.12^e^	17.83 ± 0.14^g^	16.24 ± 0.06^f^	60
Cys	Bitter/sweet/sulfur (−)	0.91 ± 0.04^abc^	0.84 ± 0.06^ab^	0.95 ± 0.03^bc^	0.99 ± 0.02^c^	0.99 ± 0.02^c^	0.83 ± 0.07^a^	0.88 ± 0.05^abc^	0.94 ± 0.06^abc^	0.87 ± 0.02^ab^	ND
Val^#^	Sweet/bitter (−)	6.13 ± 0.09^ab^	5.77 ± 0.16^a^	6.41 ± 0.09^b^	6.39 ± 0.17^b^	6.09 ± 0.13^ab^	6.20 ± 0.33^ab^	6.14 ± 0.09^ab^	8.26 ± 0.14^d^	7.25 ± 0.35^c^	40
Met^#^	Bitter/sweet/sulfur (−)	1.72 ± 0.05^ab^	1.61 ± 0.10^a^	1.92 ± 0.08^c^	1.76 ± 0.12^abc^	1.84 ± 0.09^bc^	1.70 ± 0.04^ab^	1.84 ± 0.07^bc^	2.48 ± 0.06^e^	2.18 ± 0.04^d^	30
Ile^#^	Bitter (−)	3.34 ± 0.22^a^	3.37 ± 0.17^ab^	3.87 ± 0.12^c^	3.84 ± 0.09^c^	3.82 ± 0.04^c^	3.54 ± 0.14^abc^	3.67 ± 0.18^bc^	5.48 ± 0.13^e^	4.87 ± 0.01^d^	90
Leu^#^	Bitter (−)	5.72 ± 0.06^ab^	5.53 ± 0.17^a^	6.11 ± 0.12^cd^	6.17 ± 0.18^d^	5.90 ± 0.17^abcd^	5.75 ± 0.08^abc^	5.95 ± 0.15^bcd^	8.49 ± 0.29^f^	7.67 ± 0.07^e^	190
Tyr	Bitter (−)	4.98 ± 0.27^a^	5.21 ± 0.16^ab^	5.77 ± 0.16^bc^	5.57 ± 0.41^bc^	5.56 ± 0.16^bc^	5.71 ± 0.22^bc^	5.35 ± 0.16^abc^	5.78 ± 0.24^c^	5.52 ± 0.08^abc^	ND
Phe^#^	Bitter (−)	3.22 ± 0.14^abc^	2.99 ± 0.08^b^	3.40 ± 0.10^cd^	3.29 ± 0.17^bc^	3.63 ± 0.18^d^	3.21 ± 0.11^bc^	2.82 ± 0.15^a^	5.24 ± 0.11^f^	4.46 ± 0.03^e^	90
Lys^#^	Sweet/bitter (−)	20.61 ± 0.03^ab^	22.16 ± 0.17^de^	21.17 ± 0.29^bc^	21.31 ± 0.30^c^	20.11 ± 0.32^a^	22.23 ± 0.35^e^	21.13 ± 0.17^bc^	23.70 ± 0.13^f^	21.62 ± 0.34^cd^	50
His	Bitter (−)	221.96 ± 0.90^f^	227.05 ± 0.65^g^	216.61 ± 0.64^e^	230.65 ± 0.56^h^	211.85 ± 0.39^d^	227.96 ± 0.63^g^	192.10 ± 0.60^b^	198.92 ± 0.77^c^	185.56 ± 47^a^	20
Arg	Sweet/bitter (+)	5.82 ± 0.31^ab^	6.56 ± 0.11^cde^	6.12 ± 0.09^abc^	6.19 ± 0.11^bcd^	5.59 ± 0.45^a^	7.09 ± 0.22^e^	6.32 ± 0.08^bcd^	6.65 ± 0.32^cde^	6.73 ± 0.03^de^	50
Pro*	Sweet/bitter (+)	35.45 ± 0.22^e^	33.74 ± 0.18^c^	34.514 ± 0.11^d^	35.94 ± 0.18^f^	33.99 ± 0.09^c^	32.57 ± 0.32^bc^	32.16 ± 0.14^ab^	35.19 ± 0.20^e^	32.07 ± 0.13^a^	300
Subtotal		396.94	419.54	427.46	439.88	412.05	418.48	408.03	464.44	421.02	
Taste amino acid acids (%)		28.33	30.64	33.76	32.48	33.08	29.80	37.01	39.98	38.66	

Amino acid types	Taste	Treatment process									Threshold (mg/100 g)
		B_0_	B_1_	B_2_	B_3_	B_4_	B_5_	B_6_	B_7_	B_8_	
Asp*	Umami/sour (+)	1.21 ± 0.04^a^	1.28 ± 0.03^a^	1.47 ± 0.03^b^	1.24 ± 0.05^a^	1.22 ± 0.03^a^	1.22 ± 0.02^a^	1.54 ± 0.04^b^	4.81 ± 0.04^c^	4.89 ± 0.04^c^	3
Thr^#^	Sweet (+)	20.49 ± 0.24^c^	22.02 ± 0.28^e^	19.84 ± 0.39^b^	21.26 ± 0.24^d^	15.55 ± 0.28^a^	23.60 ± 0.08^g^	22.59 ± 0.12^f^	26.13 ± 0.08^h^	21.32 ± 0.08^d^	260
Ser*	Sweet (+)	11.16 ± 0.28^b^	12.54 ± 0.03^c^	13.63 ± 0.12^d^	12.19 ± 0.25^c^	9.51 ± 0.08^a^	15.33 ± 0.29^f^	14.41 ± 0.08^e^	19.34 ± 0.07^g^	15.15 ± 0.12^f^	150
Glu*	Umami/sour (+)	3.83 ± 0.04^a^	32.66 ± 0.16^d^	43.17 ± 0.13^f^	31.39 ± 0.18^c^	26.44 ± 0.19^b^	41.18 ± 0.08^e^	43.21 ± 0.03^f^	87.77 ± 0.25^h^	68.31 ± 0.49^g^	5
Gly*	Sweet (+)	110.20 ± 0.36^b^	129.92 ± 0.13^e^	147.50 ± 0.12^h^	125.81 ± 0.23^d^	95.06 ± 0.15^a^	143.50 ± 0.08^g^	139.45 ± 0.45^f^	148.65 ± 0.14^i^	113.29 ± 0.08^c^	130
Ala*	Sweet (+)	18.31 ± 0.20^b^	20.85 ± 0.02^d^	25.66 ± 0.02^e^	19.34 ± 0.08^c^	14.58 ± 0.55^a^	28.76 ± 0.49^g^	26.33 ± 0.04^ef^	34.21 ± 0.49^h^	26.53 ± 0.41^f^	60
Cys	Bitter/sweet/sulfur (−)	1.98 ± 0.03^b^	2.24 ± 0.05^d^	2.10 ± 0.04^c^	2.07 ± 0.03^bc^	1.47 ± 0.03^a^	2.40 ± 0.04^e^	2.36 ± 0.02^e^	2.80 ± 0.03^f^	1.99 ± 0.06^b^	ND
Val^#^	Sweet/ bitter (−)	12.25 ± 0.26^b^	13.46 ± 0.12^c^	13.55 ± 0.03^c^	12.11 ± 0.24^b^	9.36 ± 0.04^a^	13.96 ± 0.03^d^	13.52 ± 0.24^c^	18.87 ± 0.07^f^	14.50 ± 0.27^e^	40
Met^#^	Bitter/sweet/sulfur (−)	3.28 ± 0.08^b^	3.66 ± 0.03^cd^	3.47 ± 0.07^bc^	3.55 ± 0.04^c^	2.32 ± 0.07^a^	3.85 ± 0.13^de^	4.12 ± 0.04^f^	5.15 ± 0.17^g^	3.90 ± 0.03^e^	30
Ile^#^	Bitter (−)	6.47 ± 0.03^b^	6.98 ± 0.03^c^	7.01 ± 0.04^c^	6.89 ± 0.17^c^	4.97 ± 0.14^a^	7.29 ± 0.07^d^	7.41 ± 0.02^d^	10.87 ± 0.04^f^	8.49 ± 0.13^e^	90
Leu^#^	Bitter (−)	9.40 ± 0.03^b^	10.14 ± 0.14^c^	10.00 ± 0.30^c^	9.48 ± 0.13^b^	7.20 ± 0.06^a^	10.66 ± 0.14^d^	10.67 ± 0.23^d^	15.73 ± 0.22^f^	12.63 ± 0.18^e^	190
Tyr	Bitter (−)	7.55 ± 0.06^c^	8.25 ± 0.22^d^	7.13 ± 0.03^b^	8.18 ± 0.13^d^	5.18 ± 0.03^a^	9.07 ± 0.03^e^	9.22 ± 0.45^e^	9.86 ± 0.04^f^	8.37 ± 0.12^d^	ND
Phe^#^	Bitter (−)	4.52 ± 0.03^c^	5.02 ± 0.07^d^	4.26 ± 0.03^b^	4.92 ± 0.03^d^	2.98 ± 0.08^a^	5.91 ± 0.06^e^	5.81 ± 0.06^e^	8.55 ± 0.03^g^	7.55 ± 0.07^f^	90
Lys^#^	Sweet/bitter (−)	25.11 ± 0.08^b^	26.00 ± 0.28^c^	27.58 ± 0.26^d^	25.03 ± 0.36^b^	19.82 ± 0.44^a^	31.40 ± 0.08^f^	29.61 ± 0.07^e^	35.05 ± 0.04^g^	27.61 ± 0.31^d^	50
His	Bitter (−)	247.11 ± 0.40^f^	257.65 ± 0.43^g^	266.19 ± 0.59^h^	229.05 ± 0.85^e^	197.36 ± 0.45^b^	219.06 ± 0.44^c^	224.22 ± 0.77^d^	229.67 ± 0.83^e^	190.75 ± 0.98^a^	20
Arg	Sweet/ bitter (+)	9.84 ± 0.12^b^	10.56 ± 0.08^c^	11.10 ± 0.06^d^	10.71 ± 0.03^c^	10.71 ± 0.06^a^	14.09 ± 0.05^g^	12.04 ± 0.04^f^	14.13 ± 0.08^g^	11.49 ± 0.03^e^	50
Pro*	Sweet/ bitter (+)	24.31 ± 0.02^c^	25.87 ± 0.20^e^	27.33 ± 0.09^g^	25.56 ± 0.08^d^	19.59 ± 0.11^a^	26.62 ± 0.04^f^	25.39 ± 0.10^d^	28.53 ± 0.02^h^	23.34 ± 0.06^b^	300
Subtotal		517.03	589.10	630.99	548.79	440.59	597.90	591.90	700.14	560.11	
Taste amino acid aaaacidacids (%)		32.69	37.88	41.01	39.27	37.77	42.92	42.29	46.18	44.90	

Note

*: indicates taste amino acid; #: indicates essential amino acid; +: indicates good taste; ‐: indicates bad taste.

A_0_–A_8_: no adding; cooking wine; cooking wine + salt; cooking wine + chive; cooking wine + ginger; cooking wine + garlic; cooking wine + pepper; cooking wine + soy sauce; all seasonings.

B_0_–B_8_: no adding; cooking wine; cooking wine + salt; cooking wine + chive; cooking wine + ginger; cooking wine + garlic; cooking wine + pepper; cooking wine + soy sauce; all seasonings.

All seasonings represented cooking wine + salt + chive + ginger + garlic + pepper + soy sauce.

Different superscript letters (a–i) in the same column indicate a significant difference (*p* < .05).

Abbreviation: ND, no determined.

### 3.5 Effects of FFS process on the content of FAAs

Maillard reaction between FAAs and sugars during cooking is essential for the development of desirable meaty aromas (Madruga et al., 2010). FAAs contents of B_0_–B_8_ were 517.03, 589.10, 630.99, 548.79, 440.59, 597.90, 591.90, 700.14, and 560.11 mg/100 g, respectively, among FAAs, the flavor amino acids accounting for 32.69, 37.88, 41.01, 39.27, 37.77, 42.92, 42.29, 46.18, and 44.90%, respectively (Table 3). FAAs contents of FFS samples were higher than that of FS samples, one possible reason was that the moisture contents of samples were greatly decreased during FFS (Li, Tu, Sha, et al., 2016; Li, Tu, Zhang, et al., 2016). Previous report also confirmed cooking did, in general, significantly increased the contents of amino acids compared with raw fish species (Erkan et al., 2010). The order of FAAs contents and percentages of flavor amino acids in grass carp was FFS > FS > fresh one. The main reasons were (a) the dissolution of amino acids was promoted by soaking treatment together with fast permeation of amino acids in soy sauce into fish meat. (b) FFS treatment accelerated the degradation of protein in grass carp tissues.

Free amino acids consumption was occurred in MR between FAAs and reducing sugars (Pei et al., 2014). The total FAAs contents of FFS were higher than that of FS (Table 3). Glu was recognized as main umami amino acids in FFS process and its contents in B_0_ were higher than that in A_0_, TAV of Glu was higher than 5 in FFS samples (B_1_–B_8_). TAV of Asp was higher than 1 in FFS with cooking wine and soy sauce soaking and FFS with all seasonings. TAV of Gly was also higher than 1 in most FFS samples (Table 5), indicating that FFS had an important contribution to the taste compounds of grass carp .

### 3.6 Effects of SS process on the content of FAAs

Free amino acids contents of C_0_–C_3_ were 598.05, 622.08, 697.66, and 585.79 mg/100 g, respectively, among them, flavor amino acids accounted for 44.50%, 47.30%, 49.99%, and 50.09%, respectively. The proportion of flavor amino acids in Shanghai smoked fish was the highest (Table 4). The main reasons were FS accelerated the oxidation and degradation of proteins (Li, Tu, Sha, et al., 2016; Li, Tu, Zhang, et al., 2016). Moreover, SS treatment was beneficial to the dissolution of FAAs, as well as crisp crust formation after FFS treatment promoted soaking solution penetrated into grass carp meat. Asp and Glu contents (TAV > 14) of in SS were higher that in FS and FFS (Table 5). Most of bitter FAAs such as Cys, Met, Tyr, and His decreased significantly in SS process. His contents were the highest among bitter amino acids in SS, which tasted bitter but could enhance the flavor effect and help to form the “meat flavor” characteristics of some aquatic products (Song, Zhang, et al., 2018; Song, Wang, et al., 2018). Therefore, SS treatment could improve the contents of FAAs and sweet/umami amino acids, significantly reducing the content of most bitter amino acids.

**Table 4:** Contents of free amino acids in second soaking for preparing Shanghai smoked fish (mg/100 g)

Amino acid types	Taste	C_0_	C_1_	C_2_	C_3_	Threshold (mg/100 g)
Asp*	Umami/sour (+)	5.54 ± 0.04^a^	6.76 ± 0.04^b^	9.20 ± 0.03^d^	8.19 ± 0.03^c^	3
Thr^#^	Sweet (+)	22.13 ± 0.14^c^	20.94 ± 0.04^b^	24.93 ± 0.13^d^	19.87 ± 0.06^a^	260
Ser*	Sweet (+)	15.80 ± 0.03^a^	16.20 ± 0.08^b^	20.39 ± 0.03^d^	16.49 ± 0.12^c^	150
Glu*	Umami/sour (+)	73.14 ± 0.04^a^	91.28 ± 0.51^b^	128.11 ± 0.25^d^	107.32 ± 0.15^c^	5
Gly*	Sweet (+)	119.85 ± 0.22^b^	126.78 ± 0.72^c^	128.89 ± 0.08^d^	108.88 ± 0.03^a^	130
Ala*	Sweet (+)	26.33 ± 0.03^a^	28.19 ± 0.04^b^	34.80 ± 0.55^d^	29.31 ± 0.03^c^	60
Cys	Bitter/sweet/sulfur (−)	2.21 ± 0.03^c^	1.87 ± 0.06^b^	2.23 ± 0.08^c^	1.66 ± 0.04^a^	ND
Val^#^	Sweet/bitter (−)	15.56 ± 0.61^a^	15.97 ± 0.04^a^	18.77 ± 0.05^b^	15.74 ± 0.21^a^	40
Met^#^	Bitter/sweet/sulfur (−)	4.33 ± 0.04^b^	3.89 ± 0.04^a^	5.07 ± 0.04^c^	3.88 ± 0.03^a^	30
Ile^#^	Bitter (−)	9.09 ± 0.05^a^	9.28 ± 0.24^a^	11.69 ± 0.21^c^	9.88 ± 0.03^b^	90
Leu^#^	Bitter(−)	13.60 ± 0.04^a^	13.58 ± 0.34^a^	17.36 ± 0.26^c^	14.36 ± 0.24^b^	190
Tyr	Bitter (−)	8.55 ± 0.24^b^	6.84 ± 0.07^a^	9.53 ± 0.03^c^	6.60 ± 0.03^a^	ND
Phe^#^	Bitter (−)	7.59 ± 0.44^b^	6.46 ± 0.04^a^	10.04 ± 0.06^c^	7.56 ± 0.09^b^	90
Lys^#^	Sweet/bitter (−)	28.21 ± 0.06^b^	27.89 ± 0.04^a^	34.98 ± 0.07^c^	27.75 ± 0.07^a^	50
His	Bitter (−)	208.75 ± 0.80^c^	209.30 ± 0.93^c^	199.28 ± 0.90^b^	173.49 ± 0.82^a^	20
Arg	Sweet/bitter (+)	11.91 ± 0.03^c^	11.77 ± 0.03^b^	14.98 ± 0.06^d^	11.57 ± 0.06^a^	50
Pro*	Sweet/bitter (+)	25.46 ± 0.08^c^	25.06 ± 0.07^b^	27.41 ± 0.05^d^	23.23 ± 0.04^a^	300
Subtotal		598.05	622.08	697.66	585.79	
Taste amino acids (%)		44.50	47.30	49.99	50.09	

Note

*: taste amino acid; #: essential amino acid; +: indicates good taste; ‐: indicates bad taste; ND: no determined. C_0_‐C_3_: all seasonings + plant oil; all seasonings + plant oil + sugar; all seasonings + plant oil + sugar +soy sauce; all seasonings + plant oil + sugar + soy sauce + five‐spice powder. All seasonings represented cooking wine + salt + chive + ginger + garlic + pepper + soy sauce. Different superscript letters in the same column indicate a significant difference (*p* < .05).

**Table 5:** Taste activity value (TAV) of amino acids of Shanghai smoked fish (mg/100 g)

Samples	Asp	Ser	Glu	Gly	Ala	Pro
A_0_	0.19	0.03	0.33	0.47	0.14	0.12
A_1_	0.21	0.03	4.22	0.46	0.13	0.11
A_2_	0.21	0.04	4.68	0.53	0.18	0.12
A_3_	0.21	0.04	4.73	0.51	0.17	0.12
A_4_	0.22	0.04	4.20	0.50	0.17	0.11
A_5_	0.19	0.03	4.66	0.42	0.15	0.11
A_6_	0.23	0.04	6.08	0.52	0.24	0.11
A_7_	0.48	0.06	10.76	0.53	0.30	0.12
A_8_	0.54	0.05	8.29	0.49	0.27	0.11
B_0_	0.40	0.07	0.77	0.85	0.31	0.08
B_1_	0.43	0.08	6.53	1.00	0.35	0.09
B_2_	0.49	0.09	8.63	1.13	0.43	0.09
B_3_	0.41	0.08	6.28	0.97	0.32	0.09
B_4_	0.41	0.06	5.29	0.73	0.24	0.07
B_5_	0.41	0.10	8.24	1.10	0.48	0.09
B_6_	0.51	0.10	8.64	1.07	0.44	0.08
B_7_	1.60	0.13	17.55	1.14	0.57	0.10
B_8_	1.63	0.10	13.66	0.87	0.44	0.08
C_0_	1.85	0.11	14.63	0.92	0.44	0.08
C_1_	2.25	0.11	18.26	0.98	0.47	0.08
C_2_	3.07	0.14	25.62	0.99	0.58	0.09
C_3_	2.73	0.11	21.46	0.84	0.49	0.08

Note

A_0_–A_8_: no adding; cooking wine; cooking wine + salt; cooking wine + chive; cooking wine + ginger; cooking wine + garlic; cooking wine + peppe and cooking wine + soy sauce; all seasonings.

B_0_–B_8_: no adding; cooking wine; cooking wine + salt; cooking wine + chive; cooking wine + ginger; cooking wine + garlic; cooking wine + pepper; cooking wine + soy sauce and all seasonings.

C_0_–C_3_: all seasonings + plant oil; all seasonings + plant oil + sugar; all seasonings + plant oil + sugar + soy sauce and all seasonings + plant oil + sugar + soy sauce + five‐spice powder.

All seasonings represented cooking wine + salt + chive + ginger + garlic + pepper + soy sauce.

The delightful flavor amino acids in Shanghai smoked fish increased by 76.81% compared with fresh grass carp. The contents of His in Shanghai smoked fish were 27.94%, significantly lower than that of fresh fish in this work, which was consistent with previous report (Yue et al., 2016). The main reason was that Maillard reaction between aldehydes and His was greatly enhanced after soaking and frying (Cordoba et al., 1994). In addition, protein decomposition of grass carp tissues and lipid oxidation degradation after FFS treatment was enhanced (Wang, 2005). Therefore, FS, FSS, and SS had a synergistic effect on the improvement of earthy‐musty smell of grass carp and the increasement of total amounts of FAAs and flavor amino acid.

### 3.7 Electronic tongue analysis of Shanghai smoked fish

**FIGURE 3:**
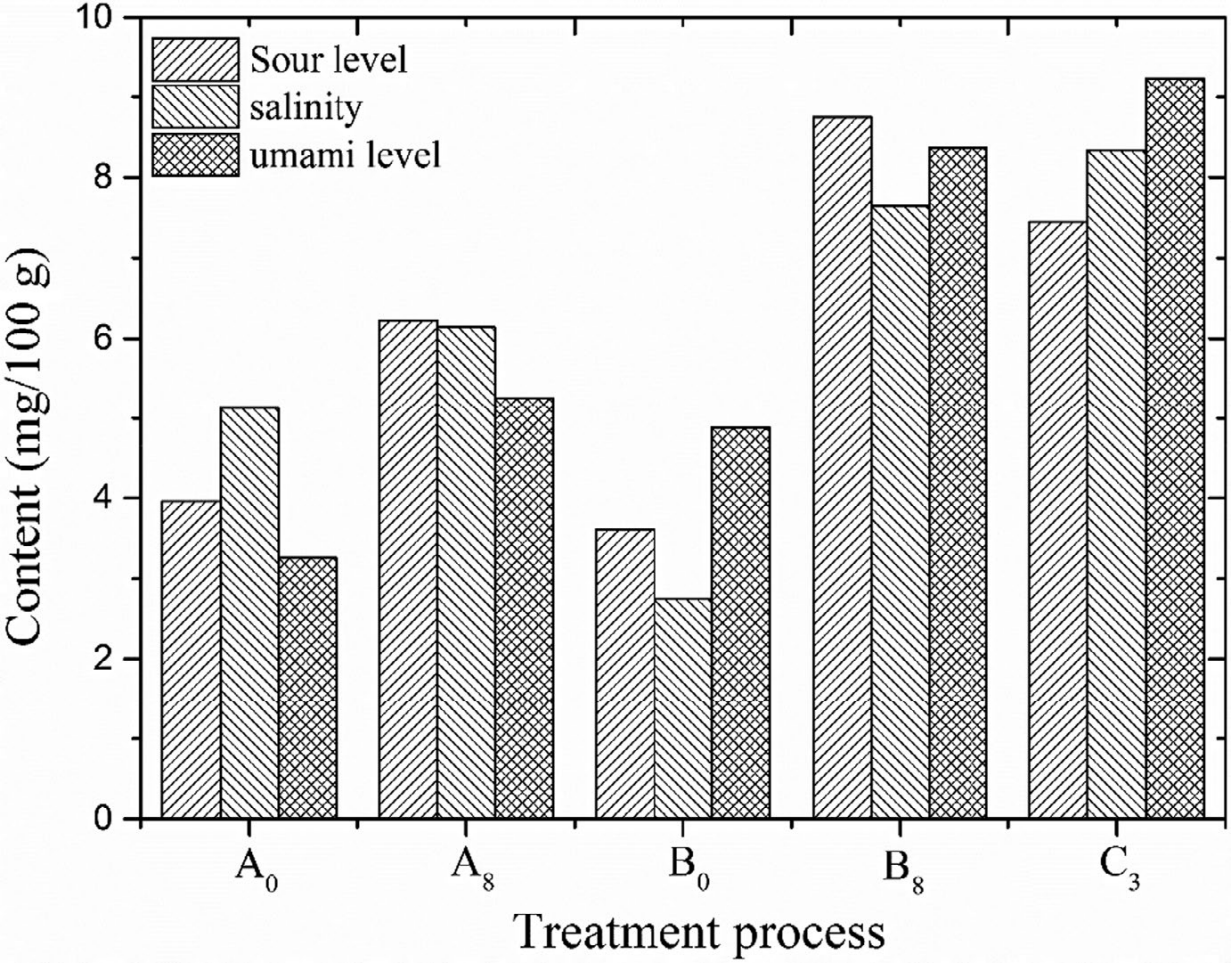
Response substances of sour level, salinity, and umami of Shanghai smoked fish (mg/100g). A_0_ and B_0_: No adding; A_8_ and B_8_: all seasonings; C_3_: All seasonings + plant oil + sugar + soy sauce + five‐spice powder. All seasonings represented cooking wine + salt + chive + ginger + garlic + pepper + soy sauce

The electronic tongue response substances of Shanghai smoked fish at different processing stages are shown in Figure 3. PCA figure (Figure 2) could well reflect the completeness of difference information of taste profile of Shanghai smoked fish. Results showed that PC1 and PC2 were 84.85% and 13.51%, as well as total contribution rate of 98.36%. There was no overlap in PCA figures of Shanghai smoked fish, which indicated that three processing stage has obvious difference in the tastes of Shanghai smoked fish. The umami level of Shanghai smoked fish was the highest, showing FS, FFS, and SS treatment synergistically contributed to the tastes of grass carp and helped to improve its umami flavor.

## 4 CONCLUSIONS

Effect of treatment process including FS, FFS, and SS on the taste compounds of Shanghai smoked fish was in detail investigated in this work. Contents of taste ATPs, FAAs, and flavor amino acids in grass carp were successively increased by FS, FFS, and SS. IMP was the main umami ATPs in the three processing stages. Glu was the main umami amino acid in FS process. Glu and Gly were the main umami flavor amino acid in FFS process. Asp and Glu had the largest effect on the nonvolatile tastes of Shanghai smoked fish. The umami level of Shanghai smoked fish reached a maximum of 9.24, indicating seasonings contributed to taste active compounds, among them, cooking wine, salt, and soy sauce also played a certain role in the tastes of grass carp. This work indicated a new processing technique of preparing Shanghai smoked fish, which not only could cover up earthy‐musty smell of grass carp, but greatly increase its umami flavor.

## CONFLICT OF INTEREST

The authors declare no conflict of interest.

## AUTHOR CONTRIBUTIONS

Chen, S.S., Zhou, Y., Zhang, H.C., involved in design and conceptualization. Zhou, Y., Zhang, H.C. involved in manuscript writing and proofreading. Wang, X.C., Zhang, H.C., and Shi, W.Z. involved in experimental work, data analysis, and interpretation. All authors have read and agreed to the published version of the manuscript.

## INFORMED CONSENT

Written informed consent was obtained from all study participants.

## ETHICAL APPROVAL

This study was approved by the ethics committee of Shanghai Ocean University.

## Data Availability

The data that support the findings of this study are available from the corresponding author upon reasonable request.
